# Raman Characterization of Fungal DHN and DOPA Melanin Biosynthesis Pathways

**DOI:** 10.3390/jof7100841

**Published:** 2021-10-07

**Authors:** Benjamin D. Strycker, Zehua Han, Aysan Bahari, Tuyetnhu Pham, Xiaorong Lin, Brian D. Shaw, Alexei V. Sokolov, Marlan O. Scully

**Affiliations:** 1 Institute for Quantum Science and Engineering, Texas A&M University, College Station, TX 77843, USA; zehuahan@tamu.edu (Z.H.); a.bahari@tamu.edu (A.B.); sokol@tamu.edu (A.V.S.); scully@tamu.edu (M.O.S.); 2 Baylor Research and Innovation Collaborative, Baylor University, Waco, TX 76704, USA; 3 Department of Microbiology, University of Georgia, Athens, GA 30602, USA; nhu.pham@uga.edu (T.P.); xiaorong.lin@uga.edu (X.L.); 4 Department of Plant Pathology and Microbiology, Texas A&M University, College Station, TX 77843, USA; bdshaw@tamu.edu

**Keywords:** Raman, SERDS, fungi, DHN, DOPA, *Aspergillus fumigatus*, *Cryptococcus neoformans*, *Aspergillus nidulans*, melanin

## Abstract

Fungal melanins represent a resource for important breakthroughs in industry and medicine, but the characterization of their composition, synthesis, and structure is not well understood. Raman spectroscopy is a powerful tool for the elucidation of molecular composition and structure. In this work, we characterize the Raman spectra of wild-type *Aspergillus fumigatus* and *Cryptococcus neoformans* and their melanin biosynthetic mutants and provide a rough “map” of the DHN (*A. fumigatus*) and DOPA (*C. neoformans*) melanin biosynthetic pathways. We compare this map to the Raman spectral data of *Aspergillus nidulans* wild-type and melanin biosynthetic mutants obtained from a previous study. We find that the fully polymerized *A. nidulans* melanin cannot be classified according to the DOPA pathway; nor can it be solely classified according to the DHN pathway, consistent with mutational analysis and chemical inhibition studies. Our approach points the way forward for an increased understanding of, and methodology for, investigating fungal melanins.

## 1. Introduction

Melanin is one of the most ancient and widespread biopigments found in almost every kingdom of life [1]. It is found in fossils of dinosaurs, early birds, nonavian theropods, and primitive cephalopods, and as such has been proposed as a biomarker for the study of evolution [1,2,3]. As a result of its ancient origin and ubiquity, melanin exhibits a wide range of composition, color, size, occurrence, and function, but may nevertheless be widely defined as a “heterogeneous polymer derived from the oxidation of phenolic or indolic compounds and subsequent polymerization of intermediate phenols and their resulting quinones” [1,4].

Melanin has gained increased attention in recent years due to its many applications in dermocosmetics [5,6,7,8], dyeing [2,5,9,10,11], environmental remediation [5,12,13], materials science [6,14,15,16,17], nanotechnology [6], and biomedicine [5,18,19,20,21,22,23]. Significantly, there is increased interest in using melanin in cancer therapy, in which it can function in multiple roles [23]. However, “a better knowledge of the physicochemical properties of the different melanins along with a better understanding of the mechanisms behind anti-cancer activity for each type of melanin is required to optimize the applications of melanin in cancer therapy” [23].

In fungi, melanin plays a multi-functional protective role against stress [12]. Melanin protects the fungal organism from ultraviolet, X-ray, gamma-ray, and particulate radiation [24,25,26]. Melanin allows certain fungi to harvest energy from ionization radiation [27,28,29]. Melanin protects against desiccation [30] and both heat and cold shock [31,32]. It helps the organism withstand chemical stressors such as hypersaline environments [33], heavy metals [34], hydrolytic enzymes [35], and reactive oxygen species (ROS) [36]. The substantial protective powers of melanin play decisive roles in the virulence of many fungal pathogens [37,38].

**Figure 1:**
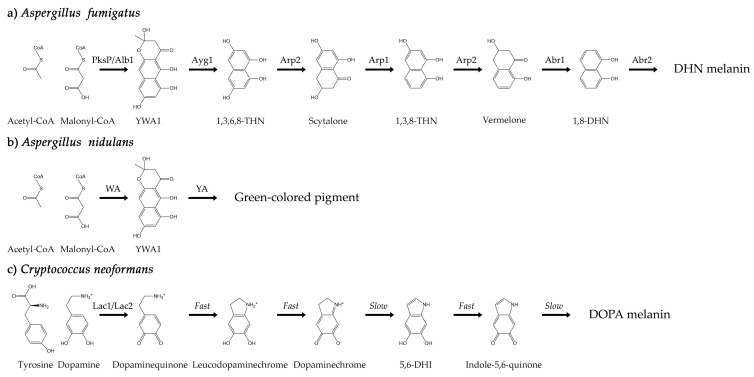
(**a**) The biosynthesis pathway of DHN melanin in *A. fumigatus* conidia. Acetyl- and malonyl-CoA are converted through a series of reactions catalyzed by six different enzymes. (**b**) The biosynthesis pathway of the green-colored pigment in *A. nidulans* conidia. Acetyl- and malonyl-CoA are converted through a series of reactions involving the WA and YA enzymes. The final product has not yet been fully characterized. (**c**) The biosynthesis pathway of DOPA melanin in *C. neoformans* cells. The initial conversion of tyrosine or dopamine to dopaminequinone is catalyzed primarily by the Lac1 enzyme. Subsequent reactions occur through a Raper–Mason scheme.

The difficulty of characterizing melanins, including fungal melanins, has long been recognized. Fungal melanins are negatively charged, hydrophobic, of high molecular weight, highly heterogeneous, insoluble in organic solvents, and resistant to chemical degradation [39,40]. Melanin is concentrated in the cell wall and bound within the cross-linked network that composes it, making elucidation of the melanin molecular structure notoriously recalcitrant to characterization [39,41]. Nevertheless, two main pathways for fungal melanin biosynthesis have been identified. One pathway begins with malonyl-CoA and leads to the polymerization of 1,8-dihydroxynapthalene (DHN), resulting in DHN-melanin. An alternative pathway begins with L-dopamine or tyrosine and leads to polymerization of dihydroxyindoles, resulting in DOPA-melanin [39]. The model organisms for the DHN and DOPA melanin biosynthesis pathways are *A. fumigatus* (Figure 1a) and *C. neoformans* (Figure 1c), respectively.

*A. fumigatus* is an opportunistic ascomycete filamentous pathogen whose airborne conidia are among the most prevalent worldwide. This fungus is responsible for 90% of aspergillosis cases, the life-threatening infection of the lungs in immunocompromised individuals [42,43,44]. Notably, the polyketide-derived DHN melanin that *A. fumigatus* synthesizes plays a key role in its virulence [45,46]. Biosynthesis of this melanin during conidiation involves six enzymes encoded by a gene cluster located on the second chromosome [47,48]. The melanin is synthesized de novo endogenously in endosomes and subsequently secreted for incorporation into the cell wall [49,50,51], anchored mostly by chitin [45].

*C. neoformans* is an opportunistic basidiomycete yeast responsible for the vast majority of fungal meningoencephalitis cases worldwide [52,53]. It enters the lungs through inhalation of spores or desiccated yeasts and, in immunocompromised individuals, often disseminates to the brain. If left untreated, the infection is lethal [52,53]. The virulence of *C. neoformans* is enhanced through both the growth of a polysaccharide capsule and melanization [54,55,56]. Unlike *A. fumigatus*, *C. neoformans* requires an exogeneous substrate such as L-dopamine or tyrosine to synthesize DOPA melanin [54]. The biosynthesis of this melanin is primarily catalyzed by a laccase enzyme encoded by *LAC1* first [57], and subsequent reactions occur through a Raper–Mason scheme [57,58,59,60,61,62]. Acid hydrolysis of melanized *C. neoformans* cells results in spherical hollow shells, called “ghosts,” composed almost entirely of melanin [63]. The same may be done with *A. fumigatus* conidia [51].

The melanin biosynthesis pathways of almost all fungi are thought to fall within the paradigms exemplified by *A. fumigatus* and *C. neoformans*. However, uncertainty about a given fungus’ melanin biosynthetic pathway can persist in the literature for years. For example, early studies in 1969 [64] and 1970 [65] indicated that the model filamentous fungus *A. nidulans*, which is closely related to the DHN-producing *A. fumigatus*, contained DOPA melanin in mycelia. Goncalves et al., concluded the same in 2012 using the melanin-overproducing mutants *mel1* and *mel2*, which exhibit brown pigmentation in mycelia [66]. In regard to conidial pigmentation, however, studies in 1999 [67] and 2001 [68] showed that the *wA* gene common to both *A. nidulans* and *A. fumigatus* encodes a polyketide synthase that can generate the naphthopyrone compound YWA1, the first precursor for conidial melanin formation in both species. Subsequent steps in conidial melanin formation for *A. nidulans* are less clear, especially when the results of chemical inhibition of biosynthesis are considered. Wheeler and Bell showed in 1988 that *A. nidulans* is unaffected by the chemical biosynthesis inhibitor tricyclazole, which inhibits conidial DHN melanin production in other species, like *A. fumigatus* [69]. Whole genome sequences show that *A. nidulans* does not carry the targeted reductases like Arp2 in the DHN melanin biosynthetic pathway [68]. It is known that the conidial melanin biosynthesis pathway of *A. nidulans* involves two genes, *wA* and *yA*, that encode the polyketide synthase WA and the laccase YA, respectively, which result in a green conidial pigment of unknown composition and structure (Figure 1b) [70]. Indeed, the uncertainty in regard to the conidial melanin of *A. nidulans* is such that, as late as 2020, it has been observed that “the involvement of the DOPA-melanin pathway in the formation of conidial pigment of this *Aspergillus* model species is still an open question” [41].

In the present work, we find that the fully polymerized *A. nidulans* melanin can be neither classified according to the DOPA pathway nor solely classified according to the DHN pathway, consistent with mutational analysis and chemical inhibition. We developed a multi-disciplinary method that takes advantage of the accumulation of melanin precursor molecules in pigment mutants of a target species and preferentially selects these melanin precursor molecules for Raman spectral measurement in vivo, thus providing a spectral “fingerprint” of each melanin precursor that can be used for further analysis and comparison [71,72]. The preferential selection of the melanin precursor molecules occurs through a resonant Raman process [73] excited by approximately 785 nm, while fluorescence signals that would otherwise disguise the information contained in the Raman spectrum are excluded through use of a Shifted Excitation Raman Difference (SERDS) technique (see [71,72] and references therein). Our spectroscopic technique has several advantages over conventional infrared spectroscopy of fungal melanins [74]: (1) It requires no sample preparation. That is, no extraction of the melanin from the cell wall matrix is required, thus preserving its native composition and structure. (2) It is insensitive to water absorption in the cells. Additionally, unlike typical infrared techniques, it can preferentially select the melanin precursor molecules for analysis through a resonant physical mechanism (3).

Since the Raman spectrum of a given molecule is indicative of its structure, melanin precursor molecules that have similar Raman spectra also have similar chemical structures. Thus, by measuring the Raman spectra of the melanin precursors contained in the pigment mutants of *A. fumigatus* and *C. neoformans*, a rough but nevertheless accurate “map” of the biosynthesis pathways of each organism can be constructed, which may be used to compare with other species. Here, we complete this “map,” and use it to elucidate the nature of melanin biosynthesis in *A. nidulans* conidia. We show that the DOPA pathway has no bearing on the production of melanin in *A. nidulans* conidia. Furthermore, while the conidial pigment produced by *A. nidulans* begins with the polyketide synthase, the final product diverges from a dichotomous classification scheme (either DOPA or DHN). Our current data are consistent with previous genetic characterization and support a new classification of melanin produced by *A. nidulans*. We show that more exact terminology is needed, especially since “research on chemical structures and biosynthetic pathways of conidial and sclerotial pigments in aspergilli is still at its infancy” [41].

We anticipate that the technique outlined below will become a powerful tool in the characterization of fungal melanins, which are attractive as low-cost and high-yield alternatives to synthetic, animal, and plant melanins [12], and have great potential for powerful and eco-friendly breakthroughs in industry and medicine.

## 2. Materials and Methods

### 2.1. Phenotypic Assay

**Figure 2:**
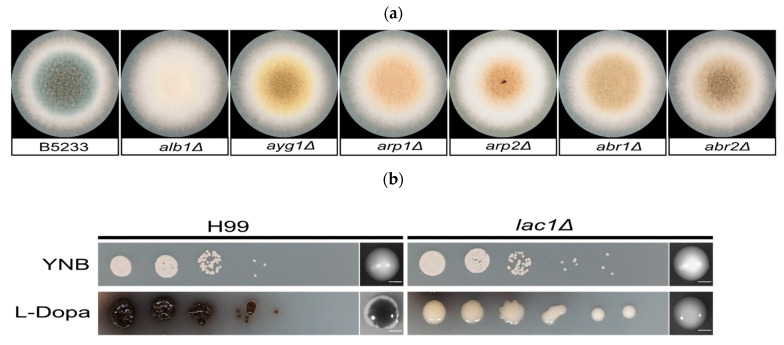
(**a**) Individual colony of the melanin mutant and the *A. fumigatus* wild-type control (B5233). A total of 10 μL of a 2 × 10^6^ cells/mL cell suspension for each strain was inoculated onto the center of an *Aspergillus* complete media (CM) plate. The plates were incubated at 30 °C for 3 days. (**b**) The laccase mutant (*lac1*∆) and the *C. neoformans* wild-type control (H99). In total, 3 μL of the serial dilutions were dropped onto YNB medium and L-Dopa media. Scale bar: 2000 µm.

*A. fumigatus* strains (wild type B5233 and melanin mutant *alb1*Δ, *ayg1*Δ, *arp1*Δ, *arp2*Δ, *abr1*Δ, *abr2*Δ [47]) from −80 °C glycerol stocks were streaked onto standard *Aspergillus* solidified complete media (CM). Cells were then cultured at 30 °C for 3 days. *A. fumigatus* mutants and wild-type spores were collected in 1 × PBS + 0.2% Tween-20. Spores were counted using a hemocytometer and diluted to a final concentration of 2 × 10^6^ cells/mL. A total of 10 μL of the spore suspension was spread onto CM plates and incubated at 30 °C. At day 2 and day 3, spores were collected and fixed in 3.7% formaldehyde. Images were acquired on day 2 and day 3 (Figure 2a).

*C. neoformans* strains (wild type H99 and *lac1*Δ [61]) from −80 °C glycerol stocks were streaked on solidified YPD media and cultured at 30 °C for 24 h. The same number of *C. neoformans* cells from each strain were suspended in sterile water, counted using a hemocytometer, and diluted to a final cell concentration of 2 × 10^6^ cells/mL. In total, 3 µL serial dilutions of these cells (10^−1^ to 10^−5^) were dropped onto YNB or L-Dopa agar media for phenotypical assay. In parallel, approximately 100 cells of H99 and *lac1*Δ were spread evenly onto YNB and L-Dopa plates and incubated in the dark at 25 °C. Images were acquired on day 4 and day 10 (Figure 2b). On day 10, cells were fixed in 3.7% formaldehyde. To produce melanin ghosts, cells were incubated in 6N HCl for 2 h at 80 °C as described in detail previously [63].

### 2.2. Spectroscopic Collection

Spectroscopic data for *A. nidulans* strains were taken from a previous work [71,75]. In that work, progeny from a cross between FGSC A773 (*wA3*) and FGSC A849 (*yA2; rodA*∆) [76] were collected in order to select strains that were both WT and *rodA*∆ for the hydrophobin and produced conidia of each of the three possible colors: wild type green, and mutants yellow and white, as previously described [77]. In the present study, only the data for the *rodA*^+^ strains were used. To collect spores, each strain was grown on a separate MM plate for seven days at 30 °C and then harvested with 1 mL sterile distilled water using a bent glass rod, for a final concentration of 1 × 10^6^ spores. Spore suspensions were stored at 4 °C and dispersed by vortexing prior to experiments.

### 2.3. SERDS Experiments

**Figure 3:**
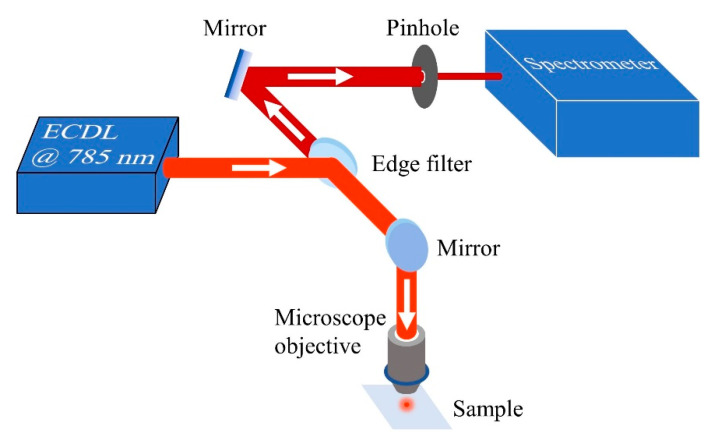
Experimental setup of shifted excitation Raman difference spectroscopy (SERDS) for spore measurements. The microscope objective focuses light emitted from a homemade external cavity diode laser (ECDL) onto the sample, generating red-shifted Raman signals that propagate backward along the laser beam path. The signals are subsequently transmitted through the edge filter and detected by the spectrometer. Refer to [71,72] for more parameter settings in the SERDS measurements.

The SERDS setup (Figure 3) consisted of a commercial confocal Raman microscope (LabRAM HR Evolution, Horiba, Kyoto, Japan) and a homemade tunable laser emitting at ~785 nm (for details refer to [71]). Before optical measurements, a small amount of spore suspension (6 μL) was deposited on a fused silica substrate (WG 41,010, Thorlabs, Newton, NJ, USA) and air-dried by evaporation for a few hours at 25 °C. The sample was then placed under the Raman microscope and excited by the tunable laser. The edge filter was used to reflect the excitation laser beam to the sample while transmitting lower-frequency (or longer-wavelength) emission signals to the spectrometer. The pinhole blocked out-of-focus signals, thus enabling signal detection only from that part of the sample directly excited by the focused laser beam.

**Figure 4:**
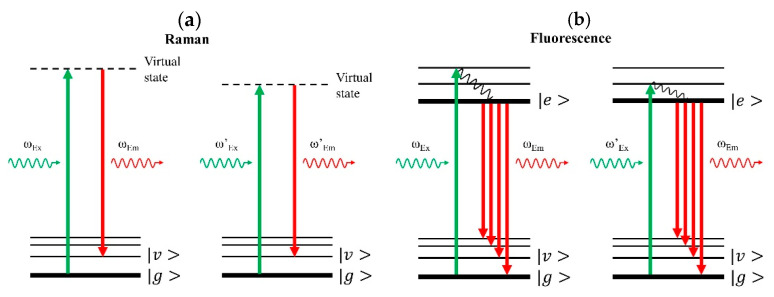
Energy diagram in Raman and fluorescence processes. |g>, |v>, and |e> denote ground state, vibrational state, and electronically excited state, respectively. The wavy lines show non-radiative transitions. Greater arrow length denotes higher frequency or higher photon energy, and vice versa. (**a**) Left panel. In Raman scattering, a molecule is pumped by a laser photon with frequency at ω_Ex_ to a virtual state and then returns to one of its vibrational states, |v>, by emitting a signal photon with lower frequency at ω_Em_. The vibrational transition matches the frequency difference between input and output photons. Right panel. For the same vibrational transition, a modified, lower pump laser frequency, ω_Ex_’, results in a corresponding signal emission at lower frequency, ω_Em_’. Thus, the frequencies of Raman features vary as the laser excitation frequency changes. (**b**) Left panel. In the fluorescence process, the molecule is upconverted to its electronically excited state, |e>, and then decays to the lowest level of the excited state for subsequent fluorescence emission, ω_Em_. Right panel. A modified pump laser frequency, ω_Ex_’, is relatively lower but still enough for the subsequent fluorescence emission. Fluorescence emission always starts from the lowest level of the excited state, and hence the frequency of fluorescence features will remain the same even if the input laser frequency varies. Consequently, slightly shifting the laser excitation frequency allows separation and detection of the pure Raman signal.

SERDS is designed to separate the Raman and fluorescence contributions in the measured spectra, which occur simultaneously as competing processes, especially in biological specimen (see Figure 4). In contrast to fluorescence (Figure 4b), the Raman response alters significantly even for a small variation in excitation frequency (Figure 4a), such that different emission frequencies give the same vibrational transition. For fluorescence, identical emission features result from different excitation frequencies. Consequently, the fluorescence contribution can be remarkably suppressed by subtracting one measured spectrum from a second measured spectrum that has been excited by a slightly shifted laser frequency. The resulting difference spectrum is then dominated by the Raman response. The pure Raman spectrum can then be reconstructed by integrating the difference spectrum according to a Raman retrieval protocol (refer to Supporting Information of [72] for more detail).

As such, in order to retrieve the pure Raman spectrum of each spore according to the above SERDS technique, two spectra with slightly different laser excitation wavelengths for each spore were recorded separately. Sample numbers and laser parameters for the spectroscopic measurements of *A. fumigatus*, *A. nidulans*, and *C. neoformans* strains are listed in Table 1, Table 2 and Table 3, respectively. All raw data for *A. fumigatus* and *C. neoformans* strains are deposited and available online at [78].

Raman and fluorescence spectra corresponding to *A. nidulans* strains were taken from previously measured raw data [71,75] and processed in an identical manner as data for *A. fumigatus* and *C. neoformans* strains, according to the protocol described in the Supporting Information of [72].

Principal component analysis (PCA) was performed on the pure, retrieved Raman spectra of selected strains using the *pca* function in Matlab [79]. The number of spectral measurements used in PCA for strains of *A. fumigatus*, *A. nidulans*, and *C. neoformans* was equal to the sample number *n* in Table 1, Table 2 and Table 3.

## 3. Results and Discussion

**Figure 5:**
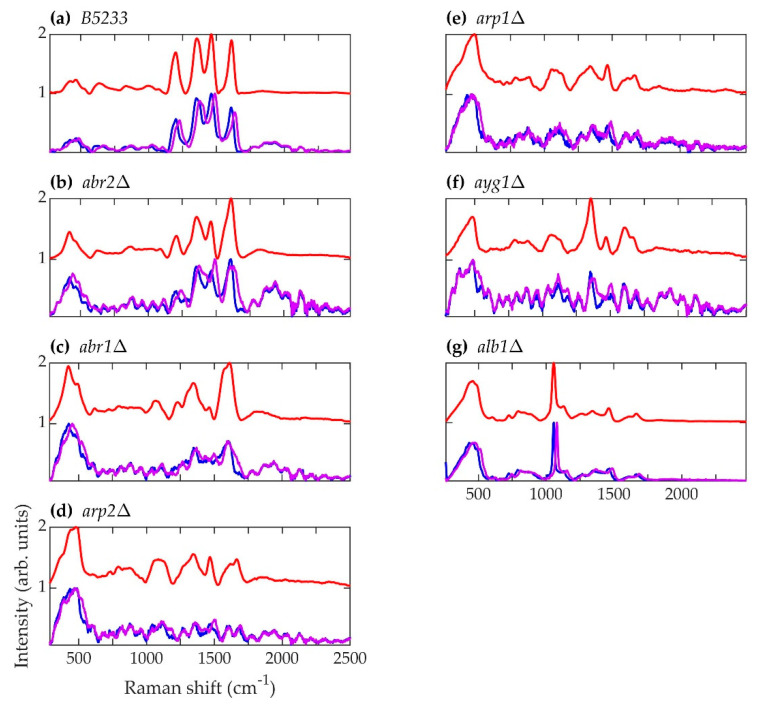
Spectra corresponding to *A. fumigatus* strains (**a**) B5233, (**b**) *abr2*Δ, (**c**) *abr1*Δ, (**d**) *arp2*Δ, (**e**) *arp1*Δ, (**f**) *ayg1*Δ, and (**g**) *alb1*Δ. Blue (~784.2 nm laser excitation) and purple (~785.8 nm laser excitation) curves are the average normalized background-subtracted raw spectra, while the red curves are average retrieved pure Raman spectra.

**Figure 6:**
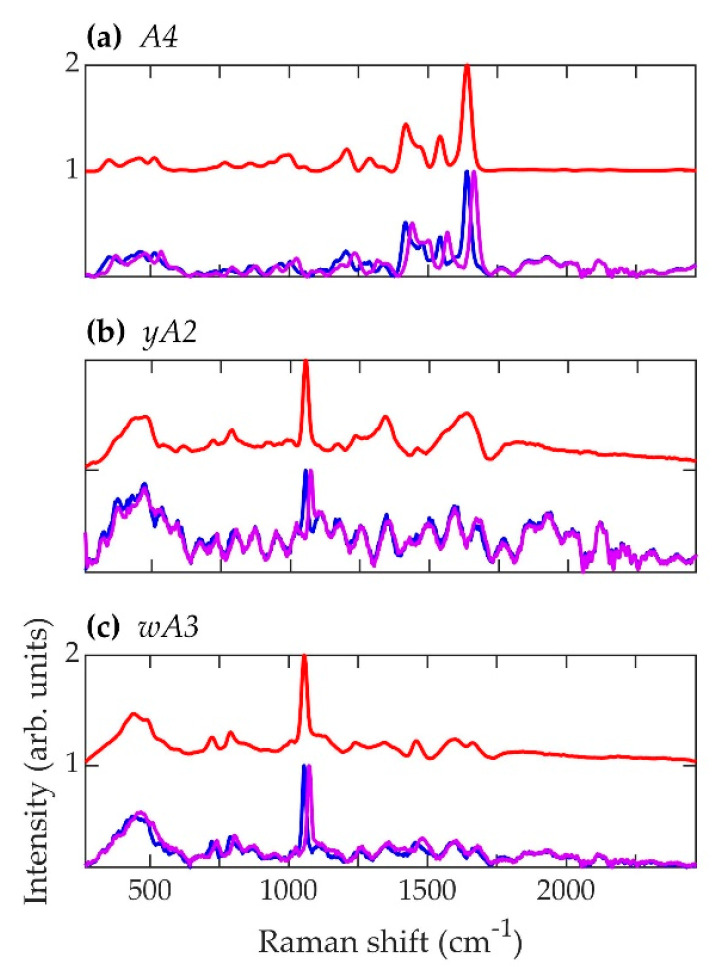
Spectra corresponding to *A. nidulans* strains (**a**) A4, (**b**) *yA2*, and (**c**) *wA3*. Blue (~784.3 nm laser excitation) and purple (~785.5 nm laser excitation) curves are the average normalized background-subtracted raw spectra, while the red curves are average retrieved pure Raman spectra.

**Figure 7:**
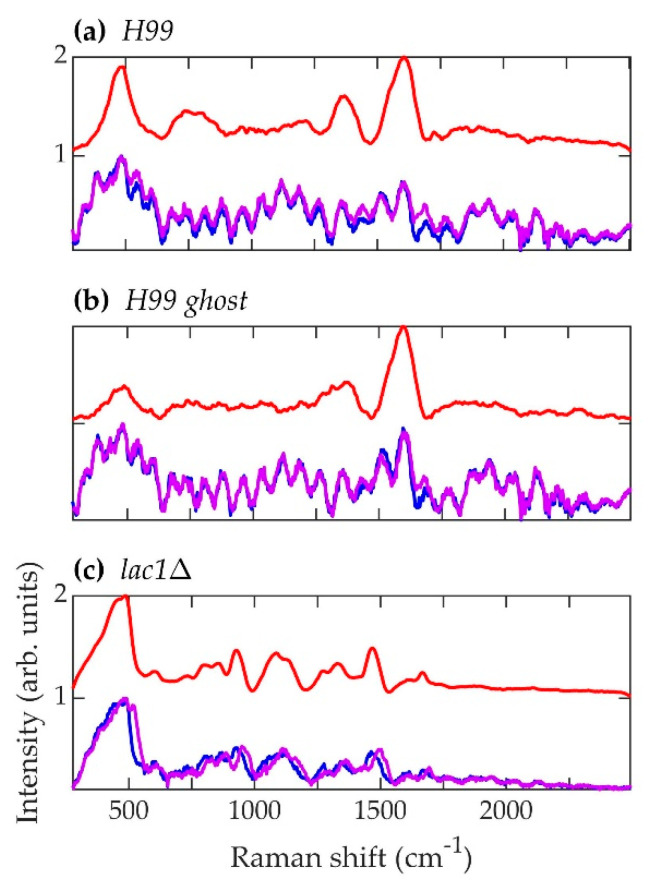
Spectra corresponding to *C. neoformans* strains (**a**) H99, (**b**) H99 ghost, and (**c**) *lac1*Δ. Blue (~784.1 nm laser excitation) and purple (~785.8 nm laser excitation) curves are the average normalized background-subtracted raw spectra, while the red curves are average retrieved pure Raman spectra.

**Figure 8:**
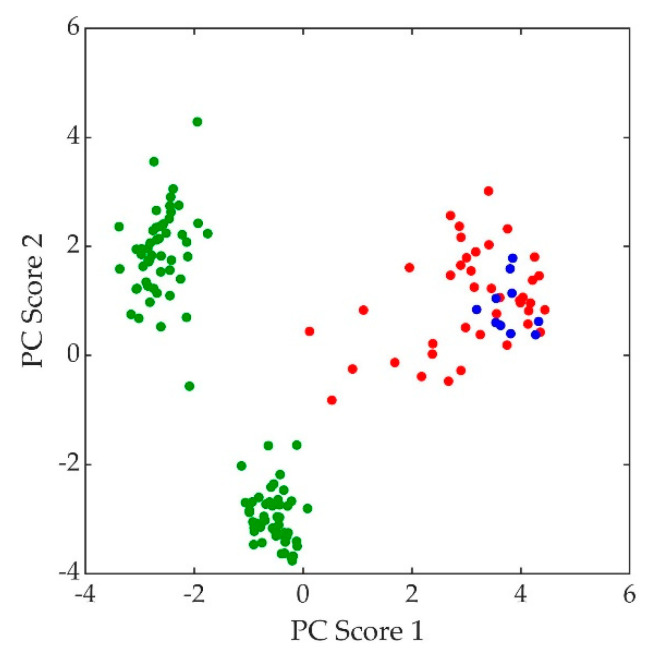
Principal component (PC) scores of *C. neoformans* strains: H99 (red), H99 ghost (blue), and *lac1*Δ (green).

The normalized, average background-subtracted raw spectra and the corresponding normalized average retrieved pure Raman spectra of *A. fumigatus*, *A. nidulans*, and *C. neoformans* strains are shown in Figure 5, Figure 6 and Figure 7, respectively. In Figure 7b, the observation that the *Cryptococcus* H99 melanin ghost exhibits an almost identical spectrum to that of an intact cell shows that the Raman spectra indeed correspond to melanin and that the polysaccharide capsule surrounding *Cryptococcus* yeast cells does not significantly contribute to the measured spectrum, because the polysaccharide capsule was completely removed in the H99 ghost sample. Additionally, see, for example, the microscopic and electron spin resonance (ESR) characterizations of *C. neoformans* melanin ghosts in [80], which show that they are composed primarily of melanin. The preferential Raman excitation of melanin results from resonance with the ~785nm laser light. This is confirmed in Figure 8, which shows the principal component scores of the H99 (red), H99 ghost (blue) and *lac1*Δ (green) strains when jointly subjected to PCA. The H99 and H99 ghost strains are overlapping, while the *lac1*Δ strain, which does not contain melanin but does have a polysaccharide capsule, is clearly distinct from the other two strains. These results confirm that melanin is the dominant contributor to the Raman spectrum of wild-type H99.

**Figure 9:**
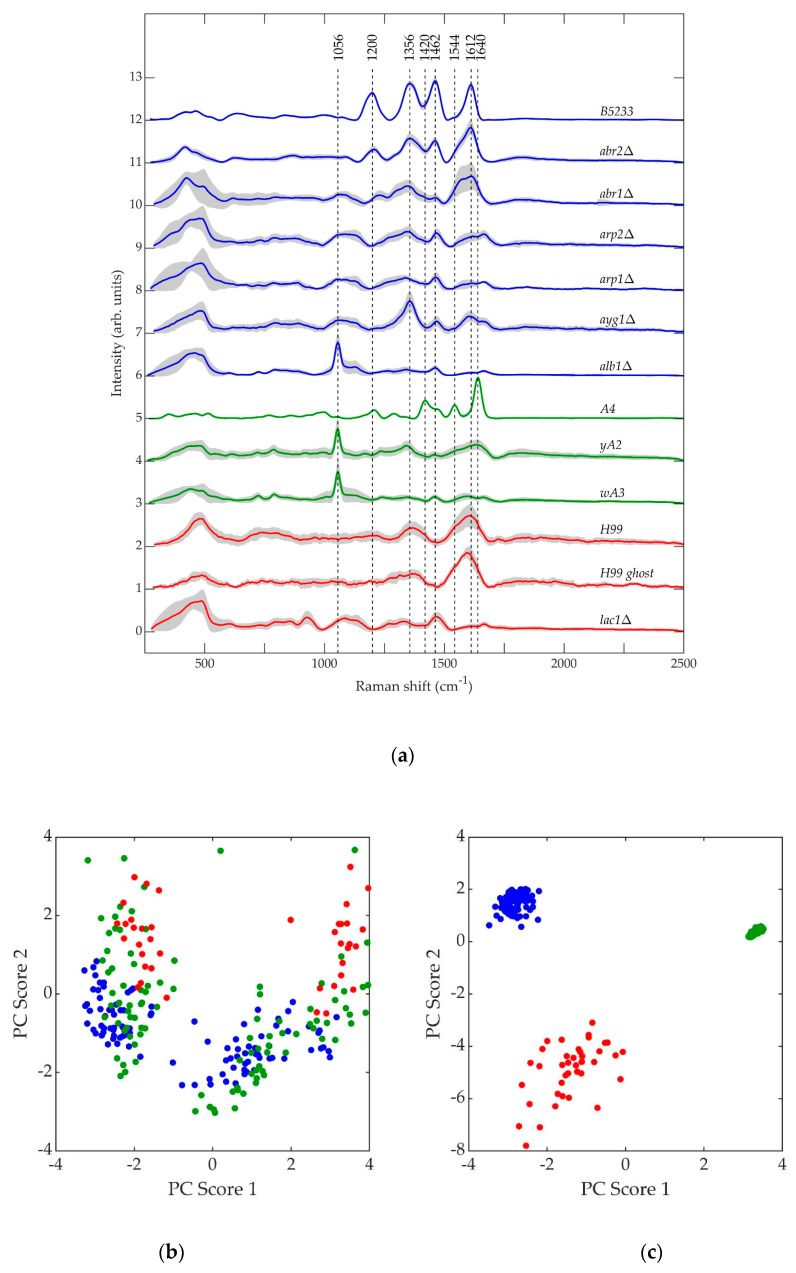
(**a**) Average normalized retrieved pure Raman spectra of *A. fumigatus* (blue), *A. nidulans* (green), and *C. neoformans* (red) strains. (**b**) Principal component scores of mutant strains at the beginning of each biosynthetic pathway: *alb1*Δ (blue), *wA3* (green), and *lac1*Δ (red). (**c**) Principal component scores of strains at the end of each biosynthetic pathway: B5233 (blue), A4 (green), and H99 (red).

Shown in Figure 9a are the average normalized retrieved pure Raman spectra of *A. fumigatus* (blue), *A. nidulans* (green), and *C. neoformans* (red) strains. The gray region surrounding each curve is the standard deviation uncertainty. Figure 9b,c shows the principal component scores of *A. fumigatus* (blue), *A. nidulans* (green), and *C. neoformans* (red) strains at the beginning and end of each respective biosynthetic pathway. The datapoints in Figure 9b,c reflect two independent PC analyses using the pure, retrieved Raman spectral data of the strains included in each respective figure. In Figure 9b, there is no clear separation of the three species if the first step of melanin biosynthesis is disrupted. In Figure 9c, there is clear separation of the three species once they are melanized. This indicates that melanin is a major contributing factor in the classification.

The primary result of interest is the striking resemblance in the pure Raman spectra of the *A. nidulans* mutant strains *wA3* and *yA2* to the *alb1*Δ strain of *A. fumigatus*, namely the prominent peak at approximately 1056 cm^−1^ in Figure 5, Figure 6 and Figure 9a. In biological tissues, this peak has been associated with lipids [81]. In the case of *A. fumigatus*, the absence in *alb1*Δ of a polyketide synthase that participates in the β-ketoacyl condensation of malonyl-CoA/acetyl-CoA most likely leads to a buildup of fatty acids. Since the regulatory action of malonyl-CoA inhibits the oxidation of fatty acids for metabolism [48], this leads to an increase in conversion of fatty acids to triglyceride lipids [82]. In short, a buildup of malonyl-CoA leads to a buildup of lipids. The same is likely to be true of *A. nidulans* strains, since in this species melanin biosynthesis also begins with malonyl-CoA [70]. The first enzymatic product of melanin biosynthesis in both *A. fumigatus* and *A. nidulans* is naphthopyrone [67,68]. It is therefore not surprising that mutant strains corresponding to early steps in the melanin biosynthesis process exhibit similar features in both species.

However, the spectral features of the *A. fumigatus* and *A. nidulans* strains diverge as the melanin biosynthesis process continues in each species. This is illustrated in Figure 9b,c. In Figure 9b, PCA of the spectral data of each strain corresponding to the beginning of each respective biosynthetic pathway shows that *A. nidulans wA3* and *A. fumigatus alb1*Δ are largely overlapped, while *C. neoformans lac1*Δ overlaps less. In Figure 9c, PCA of the spectral data of each strain corresponding to the end of each respective biosynthetic pathway shows distinct, non-overlapping groups for *A. nidulans* A4, *A. fumigatus* B5233, and *C. neoformans* H99. Indeed, the only *A. fumigatus* strain to exhibit any similarity with the *A. nidulans wA3* and *yA2* strains is *alb1*Δ, which is blocked in the very first step in the melanin metabolic pathway. The other five *A. fumigatus* mutant strains do not exhibit spectral similarity to *A. nidulans*, and in fact the fully synthesized melanins in each species likewise exhibit little similarity. We have shown in a previous work that the species *Penicillium chrysogenum* (*P. chrysogenum*) exhibits a melanin Raman spectrum (and hence melanin molecular composition and structure) of much greater similarity to *A. fumigatus* than *A. nidulans* [72]. In fact, comparison of the genes involved in melanin biosynthesis bears this out. As can be seen in Table 4, *P. chrysogenum* has the greatest genetic similarity to *A. fumigatus* [83]. Interestingly, a recent evolutionary study of melanin-related gene clusters suggested horizontal gene transfer between the *Aspergillus* and *Penicillium* genera [84].

Even though *A. fumigatus* and *A. nidulans* share the common melanin precursor naphthopyrone, it is questionable how useful it may be to classify the *A. nidulans* melanin as DHN. 1,8-dihydroxynaphthalene (DHN) is the sixth and final intermediate in the chain of biochemical reactions that leads in *A. fumigatus* from malonyl-CoA/acetyl-CoA precursors to fully polymerized melanin [39,48]. As seen in Figure 9a,c, the *A. nidulans* Raman spectra exhibit very little similarity to the *A. fumigatus* mutant strains corresponding to advanced stages of the melanin biosynthesis pathway, and *A. nidulans* is not known to form DHN. Indeed, the prominent peaks at ~1640 and ~1544 cm^−1^ in the A4 *A. nidulans* strain have previously been identified as belonging to Amides I and II [81,85], respectively. The amide compound contains nitrogen as a key component. Melanins are classified into five categories: eumelanin, pheomelanin, neuromelanin, allomelanin, and pyomelanin. Of these, only allomelanin and pyomelanin do not contain nitrogen [86,87]. DHN melanin is classified as a major subtype of allomelanin [86]. Unlike DOPA melanin, which is classified as a eumelanin [86], DHN melanin does not contain nitrogen, and this fact is used to classify fungal melanins on the basis of nitrogen content [66]. Therefore, if the prominent ~1640 and ~1544 cm^−1^ peaks in the Raman spectrum of the A4 *A. nidulans* strain do indeed arise from Amides I and II, then the *A. nidulans* conidial melanin can be classified neither as DHN nor as an allomelanin, even though it shares a common naphthopyrone precursor with *A. fumigatus*.

Consistent with the idea that *A. nidulans* conidial melanin should not be classified as DOPA melanin, the Raman spectra of the *A. nidulans* strains exhibit even less similarity to the *C. neoformans* strains than they do to the *A. fumigatus* strains (Figure 9a–c). The broad peaks in the *C. neoformans* H99 strain at ~1370 and ~1610 cm^−1^ mirror those observed in the Raman spectrum of DOPA melanin extracted from the sepia cuttlefish [88].

**Figure 10:**
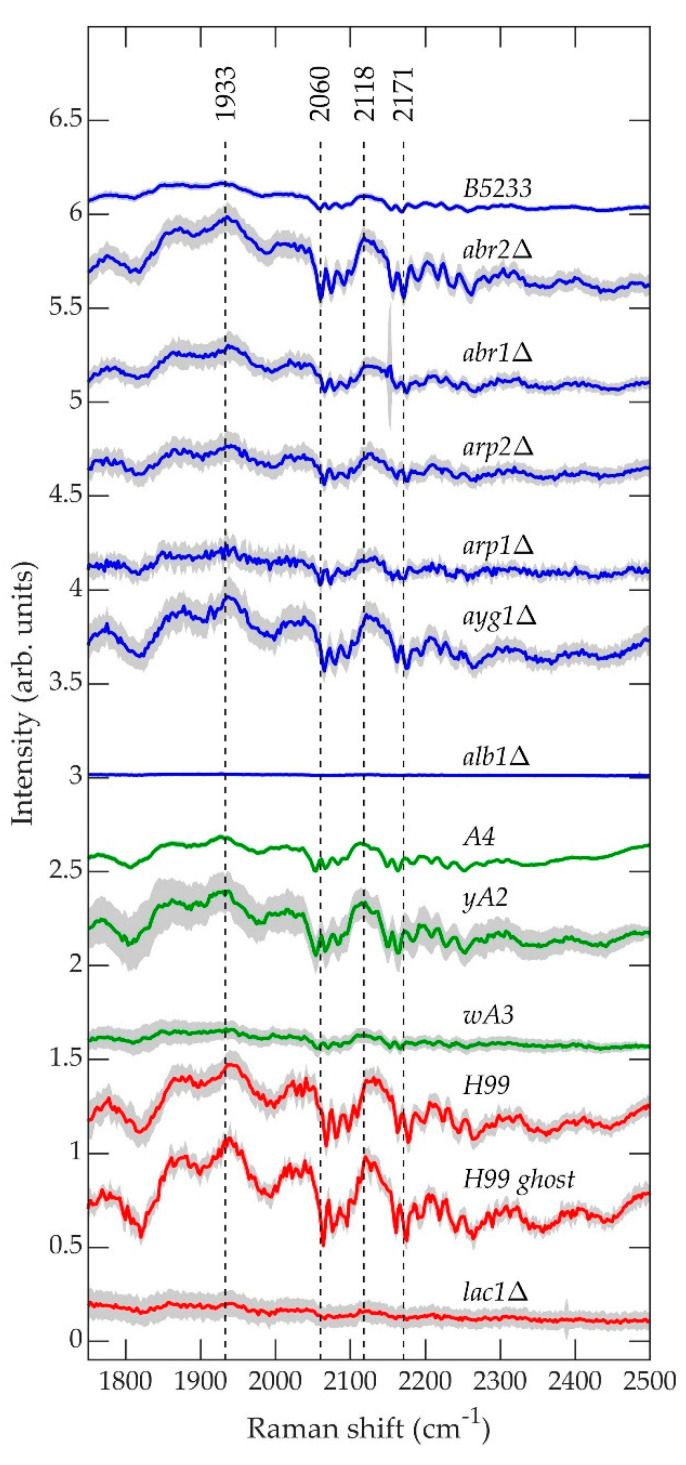
Average normalized background-subtracted raw spectra of *A. fumigatus* (blue), *A. nidulans* (green), and *C. neoformans* (red) strains in the range 1750–2500 cm^−1^.

Figure 10 contains the average normalized background-subtracted raw spectra of *A. fumigatus* (blue), *A. nidulans* (green), and *C. neoformans* (red) strains in the range 1750–2500 cm^−1^, which according to Figure 5, Figure 6 and Figure 7 are composed primarily of the fluorescence signal. The gray region surrounding each curve is the standard deviation uncertainty.

The fluorescence spectra shown in Figure 10 exhibit remarkable qualitative similarity between species, excluding the first biosynthesis pathway mutant for each, namely *A. fumigatus alb1*Δ, *A. nidulans wA3*, and *C. neoformans lac1*Δ. These three strains exhibit little or no “fine-scale fluorescence” that characterizes the spectra of the remaining strains. In a previous work, we hypothesized that the fine-scale fluorescence results from the formation of long-lived molecular cages [89,90] in the polymer matrix that constitute the cell wall, which protects the fluorescing molecules from greater levels of inhomogeneous broadening that they would otherwise experience in a liquid environment [91,92]. Figure 10 would suggest that the fine-scale fluorescence results from molecules associated with the onset of melanin biosynthesis. It is unknown what these molecules could be. Regardless, the fluorescence signal contains comparatively little information about molecular structure, and we have shown previously that it contains only a tiny fraction of the total information contained in the raw measured spectrum, which is dominated by the Raman contribution [72]. Nevertheless, the fine-scale fluorescence remains a feature of interest not only because its origin is unknown, but also because the mechanism that enables it might provide insight into quantum coherences in biological systems, such magnetoreception in birds, insects, and plants [93,94], for which coherence times are an active area of research [95,96].

## 4. Conclusions

We used a multi-disciplinary method to characterize the Raman spectra of melanin biosynthetic mutant strains of *A. fumigatus*, *C. neoformans*, and *A. nidulans* and used these spectra to elucidate the nature of the biosynthetic pathway of conidial melanin in *A. nidulans*. We find that the dissimilarity between the *C. neoformans* and conidial *A. nidulans* Raman spectra excludes the possibility that the *A. nidulans* conidial pigment may be associated with the DOPA biosynthesis pathway. We also find that the *A. nidulans* conidial melanin cannot straightforwardly be classified as belonging to the DHN pathway. While the spectral similarity of the *A. nidulans wA3* and *yA2* strains to the *A. fumigatus alb1*Δ strain confirms that they share common melanin precursors, namely malonyl-CoA/acetyl-CoA and naphthopyrone, the spectral divergence exhibited by mutant and wild-type strains further along their respective melanin biosynthetic pathways suggests that the fully polymerized melanins differ in structure and perhaps in composition. Moreover, the main ~1640 and ~1544 cm^−1^ peaks in the wild-type A4 strain of *A. nidulans* have been identified as belonging to Amides I and II [81,85], and, if this is indeed the case, the *A. nidulans* conidial melanin cannot be classified as DHN.

We anticipate that the multi-disciplinary approach detailed above will find increasing use in the characterization of fungal melanins, which have great potential for powerful and eco-friendly breakthroughs in industry and medicine.

## Figures and Tables

**Table 1:** Sample and laser parameters for *A. fumigatus* strains.

*A. fumigatus*	*n*	$\bar{\lambda_{1}}\;(\mathrm{nm})$	$\bar{\lambda_{2}}\;(\mathrm{nm})$	$\bar{\Delta \nu }\;({\mathrm{cm}}^{-1})$
*WT*	100	784.2	785.7	25.1
*abr2*Δ	100	784.2	785.9	26.9
*abr1*Δ	40	784.2	785.8	26.9
*arp2*Δ	40	784.2	785.8	26.9
*arp1*Δ	10	784.2	785.6	23.3
*ayg1*Δ	40	784.2	785.8	26.9
*alb1*Δ	100	784.2	785.6	23.3

**Table 2:** Sample and laser parameters for *A. nidulans* strains [71,75].

*A. nidulans*	*n*	$\bar{\lambda_{1}}\;(\mathrm{nm})$	$\bar{\lambda_{2}}\;(\mathrm{nm})$	$\bar{\Delta \nu }\;({\mathrm{cm}}^{-1})$
*WT*	100	784.3	785.8	25.2
*yA2*	100	784.3	785.4	18.0
*wA3*	100	784.3	785.4	18.0

**Table 3:** Sample and laser parameters for *C. neoformans* strains.

*C. neoformans*	*n*	$\bar{\lambda_{1}}\;(\mathrm{nm})$	$\bar{\lambda_{2}}\;(\mathrm{nm})$	$\bar{\Delta \nu }\;({\mathrm{cm}}^{-1})$
*H99*	40	784.1	786.0	30.5
*H99 ghost*	10	784.2	785.7	25.1
*lac1*Δ	100	784.1	785.7	27.1

**Table 4:** Melanin-related genes in *A. fumigatus* [48], *C. neoformans* [57], *A. nidulans* [70], and *P. chrysogenum* [83].

*Species*	*Known Melanin-Related Genes*
*A. fumigatus*	*pksP/alb1, ayg1, arp1, arp2, arb1, arb2*
*C. neoformans*	*LAC1 (major role), LAC2 (minor role)*
*A. nidulans*	*wA, yA*
*P. chrysogenum*	*pks17, ayg1, arp1, arp2, arb1, arb2*

## Data Availability

The raw measured spectra of *A. fumigatus* and *C. neoformans* strains can be downloaded online at [78]. The raw measured spectra of *A. nidulans* strains can be downloaded online at [75].
